# Can the use of clean energy improve the cognitive ability of the rural elderly? Evidence from clean heating policy in northern China

**DOI:** 10.1186/s12877-026-07138-x

**Published:** 2026-09-03

**Authors:** Mingcong Chen, Qingshan Ma, Qiqi Sun

**Affiliations:** 1 School of Economics and Statistics, Guangzhou University, Guangzhou, China; 2 Research Institute for Eco-Civilization, Chinese Academy of Social Sciences, Beijing, China; 3 Guangdong Institute for International Strategies, Guangdong University of Foreign Studies, Guangzhou, China

**Keywords:** Energy transition, Clean heating policies, Cognitive ability, Rural elderly people

## Abstract

The clean transformation of traditional energy sources is of great significance for the formation and development of human capital in rural areas. We use 5-wave survey from the China Health and Retirement Longitudinal Study (CHARLS) between 2011 and 2020, and based on the quasi-experiment of the "winter clean heating" policy in northern regions implemented by the Chinese government, construct a staggered difference-in-differences (DID) model to estimate the impact of large-scale clean energy use in northern rural China on cognitive impairment among the elderly. We find that the clean heating policy improved the average cognitive ability of the elderly in rural northern China by 3.3%. This policy has a positive impact on the cognitive ability of the elderly by increasing the likelihood of clean energy use in rural households and improving local air quality. Heterogeneity studies find that the "winter clean heating" policy has a more pronounced effect on elderly women with less education levels and those living with their children. Our study provides empirical evidence for the formulation of energy transition and clean heating policies in developing countries.

## Introduction

As the world's largest developing country, China has experienced rapid economic development in recent years. However, the extensive use of coal as well as other polluting energy sources has also made China one of the world's largest countries with air pollution [1, 2]. The widespread use of coal and other polluting energy sources for heating in the northern regions, particularly in winter, has led to a large amount of pollutant emissions [3–5], seriously damaging the physical health of the people [4, 6, 7]. According to the World Health Organization (WHO), about 7 million people die prematurely every year due to air pollution^1^, which is more serious in developing countries.

With the increase in life expectancy and the decline in birth and death rates, population aging has become a global demographic trend [8]. Compared with other developing countries, China's aging speed is faster [9]. This demographic shift brings pressing public health challenges, among which cognitive decline and dementia represent a rapidly growing burden. Globally, about 55 million people live with dementia, with nearly 10 million new cases annually—a number projected to rise to 152 million by 2050 [10]. The increase is particularly pronounced in developing countries, including China. In China, with 296.97 million people aged 60 and above (accounting for 21.1% of the population) by the end of 2023 [11], approximately 15.07 million older adults are estimated to live with Alzheimer's disease, a figure expected to reach 28.98 million by 2050 [12]. This rising prevalence places immense pressure on individuals, families, and societal care systems, with global costs projected to approach US$2 trillion by 2030 [13].

Fortunately, in recent years, the Chinese government has implemented a series of air pollution policies, such as the "Ten Air Pollution Regulations", "Two Control Zones", "Total Volume Control" and "Environmental Protection Inspections", which have led to a significant improvement in overall air quality. However, in northern regions, heavy pollution weather such as smog still occurs frequently in winter due to heating and other reasons [14]. In contrast, the use of clean energy sources such as electricity and natural gas for heating emits less pollution. However, due to factors such as high investment and high conversion costs [15], rural areas in northern regions still use coal for heating for a long time [4, 16]. In order to improve seasonal pollution in the northern region, the Chinese Government has launched a pilot program supported by the central financial department for clean winter heating policies in the northern region since 2017, and has set up 43 pilot cities. Targeted financial subsidies arranged by the state finance to pilot cities to promote the adoption of natural gas and other clean energy for heating in the pilot areas. This policy provides an excellent opportunity to explore the healthy effects of the transition from traditional to clean energy.

Existing literature has primarily focused on the adverse health effects of air pollution, but there is not enough attention paid to the vulnerable group of rural elderly people. Previous studies generally agreed that the large-scale emissions of particulate matter such as PM2.5 and PM10 are detrimental to the health of the population [17–19]. Fan et al. [4] found that environmental pollution caused by winter coal combustion in northern China increased mortality rates. Chen et al. [20] found that air pollution caused by winter heating reduced life expectancy in the northern region of China by 5.5 years, and also, Fan et al. [6] found that a reduction in SO2 reduced mortality. Regarding the effects of air pollution on the health of older people, Niu et al. [21] found that air pollution is one of the most important factors leading to depression in older people, but failed to effectively identify a causal relationship between air pollution and the health of older people.

As pollution worsens, the substitution relationship between clean energy and traditional energy has gradually attracted attention. Research on energy transition in developed countries started earlier, and it is generally believed that energy transition can reduce air pollution and benefit health. For example, Barreca, Clay, and Tarr [22] studied the adoption of clean energy to replace coal heating in the United States, and found that the reduction of coal use in winter led to a 1.25 percent reduction in the population mortality rate, and the infant mortality rate was reduced by 3.27 percent. Similarly, Lueken et al. [23] found that a shift from coal to clean energy generation in the US led to a reduction in heavily polluted weather and reduced health losses by at least $20bn. Scott and Scarrott [24] used data from New Zealand and found that New Zealand's policy of encouraging households to use clean energy significantly reduced pollutant emissions.

The Chinese government first introduced policies in 2010 to encourage residents to use clean energy instead of coal [16]. However, the existence of transition costs has made it difficult for residents to switch from traditional coal resources to clean energy. Until 2017, the clean heating policy supported by the central government in pilot cities has promoted the energy transition in the northern regions, especially in rural areas. Existing studies on the evaluation of clean energy heating policies mostly focus on exploring the impact of the policy on pollutant emissions, and rarely pay attention to its health effects. For example, Xue et al. [2] found that the clean heating policy can effectively mitigate air pollution, resulting in a 20.4% decrease in SO2 emissions in the northern region, but has no effect on PM2.5 emissions, and Tan et al. [16] found that the policy is effective in reducing heavy pollution in winter in the northern region. There is limited literature that examined the health effects of clean heating policies, with some studies only roughly examining the impact of these policies on mortality rates, and few studies focusing specifically on rural areas or directly investigating the impact of these policies on the health of the vulnerable group of rural elderly.

In light of this, we use the clean heating policy implemented in northern China as a starting point, and employ data from the 2011–2020 CHARLS survey. It takes advantage of the differences in the timing of policy implementation across cities, constructing a staggered DID model to accurately identify the impact of clean heating policies on the cognitive ability of the rural elderly. Before conducting the empirical study, this paper introduces Grossman's [25] health demand model and elaborates on the transmission mechanisms of the impact of clean heating policies on the health of rural elderly from the two aspects of air quality improvement and the use of clean energy, which helps to comprehensively understand the implementation effects of clean heating policies. We further analyze the heterogeneous effects of the policy. Finally, this paper provides a rough estimate of the health benefits of clean heating policies.

We find that, firstly, the implementation of clean heating policies in northern China during the winter has improved the health conditions among the rural elderly. Specifically, it has resulted in a 3.3% increase in cognitive ability among the rural elderly. Secondly, this policy has a positive impact on the cognitive ability of the elderly by increasing the probability of rural residents using clean energy and improving local air quality. Thirdly, heterogeneity analysis reveals that the policy has a more significant impact on elderly people with higher education levels and those living with their children. Therefore, it is necessary to further leverage the positive impact of clean energy use on the health of rural elderly, in order to achieve sustainable development.

Our study’s potential contributions are primarily reflected in three aspects. First, it expands the literature on the relationship between air pollution and health, as well as the evaluation of clean heating policies. Extensive research has been conducted on the relationship between air pollution and health [17–19], but there is insufficient research on how air pollution affects the health of rural elderly people who are vulnerable to external environmental factors, and the examination of cognitive function closely related to air pollution is also inadequate. While recent work has begun to evaluate the health impacts of clean heating policies—such as Liao et al. [26], who find positive effects on physical health—the focus on cognitive outcomes remains limited. The literature on the evaluation of clean heating policies has mostly examined impacts on air quality [16, 27], with only emerging attention to health, and rarely to cognition. Therefore, this paper innovatively uses the clean heating policy implemented in northern China as a starting point to explore its impact on the cognitive health of rural elderly people. This not only extends the growing policy evaluation literature exemplified by Liao et al. [26] into the domain of cognition but also provides evidence from a developing country on how environmental interventions can mitigate pollution-related cognitive decline.

Second, previous literature identifying the impact of the clean energy transition on residential health inevitably faces the issue of sample selection bias, which leads to biased measurement results [28–31]. This is because households that adopt clean energy have a strong element of self-selection [32, 33]. Based on the top-down implementation of clean heating pilot policies in China, this paper adopts the DID model, the most commonly used policy evaluation method [34], to mitigate the aforementioned issues and effectively identify the health effects of large-scale clean energy use in rural areas.

Third, this paper can provide an empirical reference for the formulation of energy transition and clean heating policies in developing countries. Energy transition in developing countries has always been a focus topic in the relevant literature, and due to the existence of transition costs, energy transition in developing countries is often difficult to promote [16, 35, 36]. This paper finds that the clean heating policy implemented in China, which is directly subsidized by central finance, can produce good results and can provide a practical reference for developing countries to promote energy transition and formulate effective clean heating policies.

The rest of the paper is organized as follows. Sect. " Policy introduction and research framework" is the policy introduction and research framework. Sect. " Research Design and Data Description" is the research design. Sect. " Basic results and robustness tests" is the baseline results and robustness tests. Sect. " Analysis of mechanisms" is the mechanism analysis. Sect. " Extensions" is extensions. Sect. " Research findings and discussions" is the conclusions and discussion.

## Policy introduction and research framework

### Introduction of the policy

In northern China, winter temperatures are generally below 0 degrees Celsius. The heating system adopted in this region is mainly based on the former Soviet Union model and was gradually completed between 1950 and 1980. Cities in the northern region enjoy unified heating service from November 15th to March 15th each year, with centralized boilers linked to residential communities and commercial buildings through the heating system [4], while the southern region does not enjoy unified heating. However, centralized heating has not been achieved in suburban and rural areas in the northern regions. Residents in rural areas often use straw, firewood, and untreated coal for heating [16, 37], which leads to large amounts of pollutant emissions in northern China during winter [20], resulting in severe seasonal pollution. In addition, the lack of awareness among rural residents about pollution prevention, combined with long-term exposure to polluted air, has caused significant harm to the health of local residents [16, 20].

**Fig. 1:**
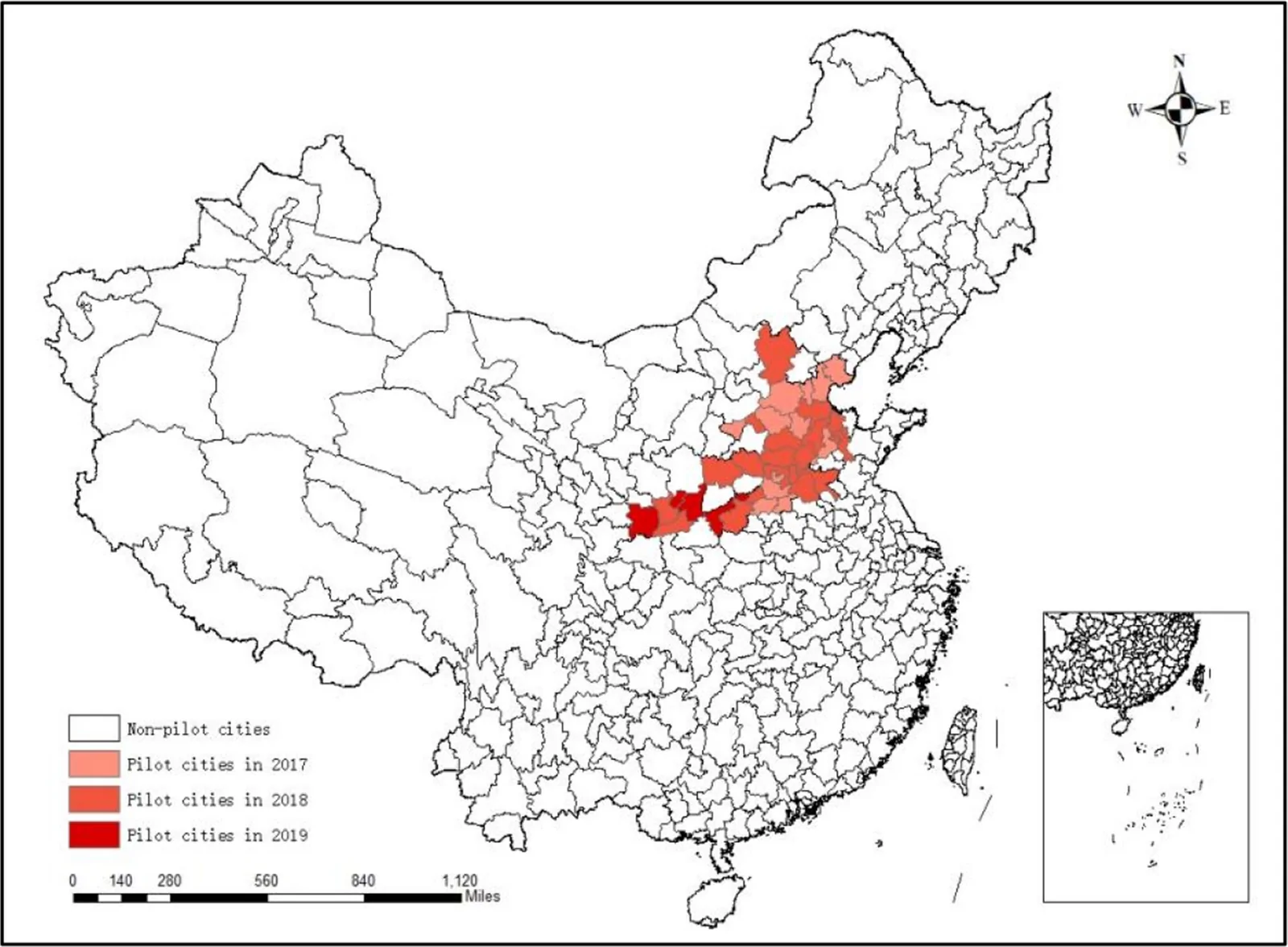
Distribution map of the clean heating pilot cities

To address the issue of increased seasonal pollutant emissions in the northern region during winter and to ensure the quality of life for residents, especially those in rural areas of northern China, the Chinese government launched a central government-funded clean heating pilot program in the northern region in 2017. In the following years, with a focus on regions such as Beijing-Tianjin-Hebei and surrounding areas, and the Fen-Wei Plain, the government continued to promote clean heating in northern China during the winter. Tailored measures were implemented to control the use of scattered coal, and 43 pilot cities were established in three batches, as shown in Fig. 1. The pilot cities are first voluntarily declared by the cities themselves, then scored by experts, and finally selected based on the ranking of scores. The scores are determined based on three factors: the local air pollution situation, local environmental protection expenditures, and related regulatory measures, and the feasibility of the technical roadmaps provided by each city. In addition, the Chinese government has formulated corresponding regulations and assessment targets for these pilot cities. Only when these cities have completed these assessment indicators within a certain time frame can they receive financial subsidies, while cities that fail to pass the assessment will have part of their subsidies deducted and canceled.

After receiving central financial subsidies, each pilot city actively promotes energy transformation work, advancing simultaneously from the "heat source side" and the "user side". According to data released in the 2021 'China Coal Management Research Report', the central government provided a total subsidy of 35.12 billion yuan for the first three batches of pilot projects, and local governments provided matching subsidies of 77.7 billion yuan. upply-side Infrastructure Investment: For the "heat source side", a significant portion of funds was directed towards large-scale projects to enable the transition. This included building and expanding natural gas pipeline networks, upgrading electrical grid capacity to handle increased demand from electric heating, and developing district heating systems based on cleaner energy sources. For the "user side", central and local matching funds were used to directly subsidize rural households for the purchase and installation of clean heating equipment, such as air-source heat pumps, efficient electric heaters, or gas boilers, facilitating the switch from coal-burning stoves [30, 31]. To ensure the affordability and sustained use of clean energy, pilot areas implemented preferential electricity and natural gas pricing policies for heating. These ongoing price subsidies were essential to offset the higher operational costs of clean energy compared to coal, preventing a return to solid fuel use. According to data from the Ministry of Ecology and Environment, as of the end of 2022, approximately 37 million rural households in China have completed clean heating reforms.

The implementation of clean heating pilot policies has reduced the cost of energy transition and promoted the large-scale use of clean energy in northern China, playing an important role in improving seasonal air pollution in the region [16]. However, the impact of this policy on the health of rural elderly people has not received much attention. Rural elderly people, as an environmentally sensitive and vulnerable group, generally have low levels of prevention awareness and are exposed to polluted air for long periods [28]. The transition from polluting energy sources like coal to clean energy sources for heating has undoubtedly improved the living environment for rural elderly people. Therefore, this paper further examines the effects and mechanisms of this policy on cognitive impairment among rural elderly people based on the clean heating policy implemented in northern China. This will contribute to a better understanding of the impact of clean heating policies and promote healthy aging.

### Research framework

#### Clean heating policies can promote cleaner energy use in households

The concept of the ‘energy ladder’ posits that households prioritize their energy choices according to factors such as affordability, cleanliness, and efficiency, as outlined by van der Kroon et al. [38]. Assuming that households have no preference between clean energy and polluting energy, the usage of the two types of energy depends on the amount of heat generated by each unit of energy, and the total amount of energy required for household heating in winter is fixed. Then, household energy consumption can be expressed by Eq. (1).

1 $$Q={aQ}_{{}_1}+{bQ}_{{}_2}$$

where Q denotes the total energy consumption for home heating, Q_1_ denotes the consumption of polluting energy, Q_2_ denotes the consumption of clean energy, and the coefficients *a* and *b* denote the substitution relationship between the two energy sources, which are fixed constants. The cost function of home heating is shown in Eq. (2).

2 $$C=min(\frac a{P_1},\frac b{P_2})$$

where *P*_*1*_ and *P*_*2*_ are the prices of polluting and clean energy sources respectively. This equation implies that household energy choices depend on the price of that energy. The clean heating policy takes a subsidized approach to clean energy, which reduces the price of clean energy use and leads to an increase in the likelihood of households using clean energy. Similarly, subsidies for clean energy can also be regarded as an increase in real income, leading to a shift in energy consumption patterns [39]. Therefore, this paper proposes Hypothesis 1.

Hypothesis 1: Clean heating policies promote clean energy use in households, which in turn contributes to the cognitive ability of rural older people.

#### Reduced air pollution promotes the health of older persons

Existing literature confirms that the implementation of clean energy heating policies can reduce air pollution emissions through energy structure transformation and technological innovation [2, 40]. Based on Grossman's [25] health demand model to explore the impact of air pollution on health. The utility of an individual in period t is shown in Eq. (3).

3 $${U}_{t}=U({H}_{t},{Z}_{t})$$

where *H*_*t*_ denotes the level of health and *Z*_*t*_ denotes the composite product. The increment of health capital can be expressed as Eq. (4):

4 $${H}_{t+1}-{H}_{t}={I}_{t}-{\delta }_{t}{H}_{t}$$

where* I*_*t*_ and *δ*_*t*_ denote health investment and the depreciation rates of health capital in period t, respectively. Building on this foundation, Cropper [41] introduced air pollution into the health capital depreciation rate function, setting it as Eq. (5):

5 $${\delta }_{t}={\delta }_{0}{e}^{{\delta }_{t}}{e}^{{\delta }_{p}}f(F)$$

where *δ*_*0*_ is the original healthy capital depreciation rate. *t* and *p* denote age and air pollution level. Age and air pollution are assumed to have constant elasticity with respect to the depreciation rate of health capital. *F* is other factors affecting the depreciation rate. Consumers' investment functions for health and other goods are shown in Eqs. (6) and (7).

6 $${I}_{t}={I}_{t}({M}_{t},{TH}_{t};{E}_{t})$$

7 $${Z}_{t}={Z}_{t}({X}_{t},{TZ}_{t};{E}_{t})$$

where M_t_ is the factor of production inputs for health that consumers can purchase. X_t_ is the production input factor for the general consumption good* Z*. *TH*_*t*_ and *TZ*_*t*_ are the production time required for *I* and *Z*, respectively. *E*_*t*_ is the stock of human capital such as education. The budget constraints are as in Eqs. (8) and (9).

8 $${\sum\nolimits_{t=0}^n}\frac{V_tM_t+V^{\prime}_tX_t}{\left(1+r\right)^t}={\sum\nolimits_{t=0}^n}\frac{W_tTW_t}{\left(1+r\right)^t}+A_0$$

9 $${TW}_{t}+{TL}_{t}+{TH}_{t}+{TZ}_{t}=\Theta$$

where *V*_*t*_ and $\mathrm{V}^{\prime}_t$ are the market prices of *M*_*t*_ and *X*_*t*_, respectively. *W*_*t*_ is the wage rate in period t, TW_t_ is the working hours in period t, *r* denotes the interest rate,* A*_*0*_ is the initial wealth, and *TL*_t_ is the sick time. Based on Eqs. (8) and (9), we can get Eq. (10).

10 $${\sum\nolimits_{t=0}^n}\frac{V_tM_t+\mathrm{V}^{\prime}_t X_t+W_t\left(TL_t+TH_t+TZ_t\right)}{\left(1+r\right)^t}={\sum\nolimits_{t=0}^n}\frac{W_t\Theta}{\left(1+r\right)^t}+A_0=R$$

The total cost of consumer investment in health can be represented as CH_t_ = V_t_M_t_ + W_t_TH_t_. The total cost of consumer investment in non-health product Z is CZ_t_ = V'_t_X_t_ + W_t_TZ_t_. Now, maximizing utility subject to the constraints of Eqs. (4), (6), (7) and (10). Given the initial health capital and depreciation rate, the following equilibrium conditions can be obtained.

11 $${\gamma }_{t}+{a}_{t}=r-{\widetilde{\pi }}_{t-1}+{\delta }_{t}$$

where $${\gamma }_{t}=\frac{{W}_{t}{G}_{t}}{{\pi }_{t-1}}$$$${G}_{t}=\frac{\partial {h}_{t}}{\partial {H}_{t}}$$_,_$${\pi }_{t-1}=\frac{\partial {CH}_{t-1}}{\partial {I}_{t-1}}$$. $${G}_{t}$$ is the marginal output of healthy capital, thus $${\gamma }_{t}$$ denotes the marginal monetary rate of return (MEC) on health investments. $${a}_{t}=\frac{\frac{{U}_{ht}}{\lambda }(1+r{)}^{t}{G}_{t}}{{\pi }_{t-1}}=\frac{{U}_{ht}{G}_{t}(1+r{)}^{t}}{\lambda {\pi }_{t-1}}$$, $${U}_{ht}=\frac{\partial U}{\partial {h}_{t}}$$ indicates the marginal utility of healthy time. λ denotes the marginal utility of wealth. a_t_ denotes the marginal utility rate of return on health investments. $${\widetilde{\pi }}_{t-1}=\frac{{\pi }_{t}-{\pi }_{t-1}}{{\pi }_{t-1}}$$.

To simplify the analysis, according to Grossman [25], ignoring the consumer goods nature of health capital and the variation in the marginal cost of health investments, and assuming that ${\widetilde{\pi }}_{t-1}=0$, Eq. (11) can be simplified to Eq. (12).

12 $${\gamma }_{t}=r+{\delta }_{t}$$

Based on Eq. (12), health demand curve (D) and health supply curve (S) can be plotted, with the health demand curve having a slope of

13 $$\frac{\partial {\gamma }_{t}}{\partial {H}_{t}}=\frac{\partial \frac{{W}_{t}{G}_{t}}{{\pi }_{t-1}}}{\partial {H}_{t}}=\frac{{W}_{t}}{{\pi }_{t-1}}\frac{\partial {G}_{t}}{\partial {H}_{t}}$$

**Fig. 2:**
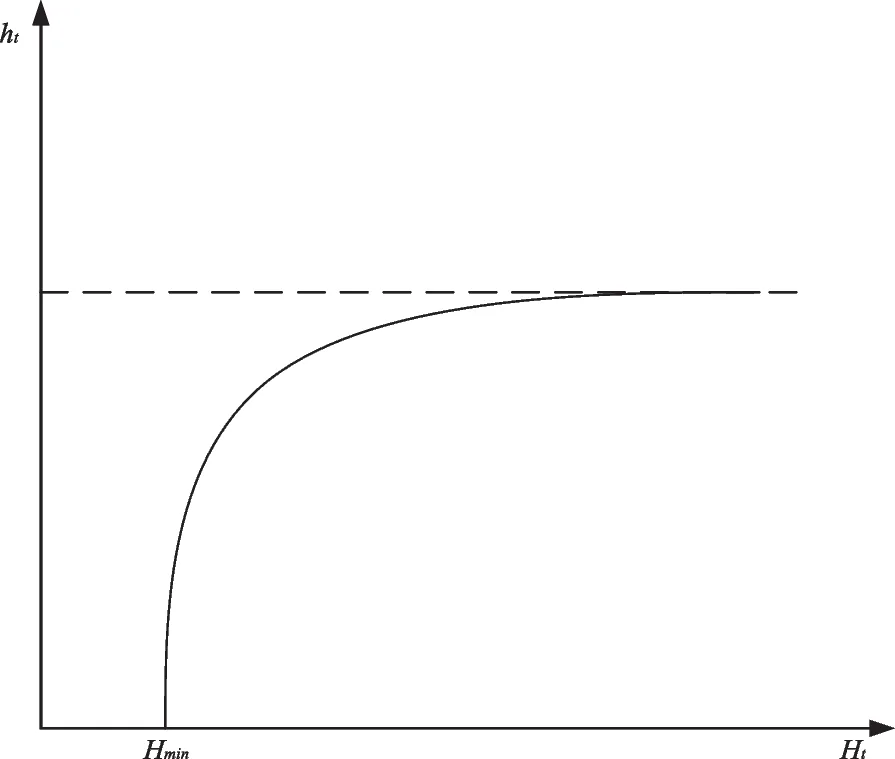
Healthy time production function

The sign of Eq. (13) depends on $\frac{\partial {G}_{t}}{\partial {H}_{t}}$, ${G}_{t}=\frac{\partial {h}_{t}}{\partial {H}_{t}}$. Since the healthy time *h*_*t*_ is finite, the relationship between *h*_*t*_ and *H*_*t*_ can be plotted as in Fig. 2 (The dotted line indicates the maximum amount of healthy time an individual can have). We can see that ${G}_{t}\ge 0$. As H_t_ increases, G_t_ gradually decreases. Therefore, $\frac{\partial {G}_{t}}{\partial {H}_{t}}<0$*.* The slope of the health demand curve (D) is negative.

**Fig. 3:**
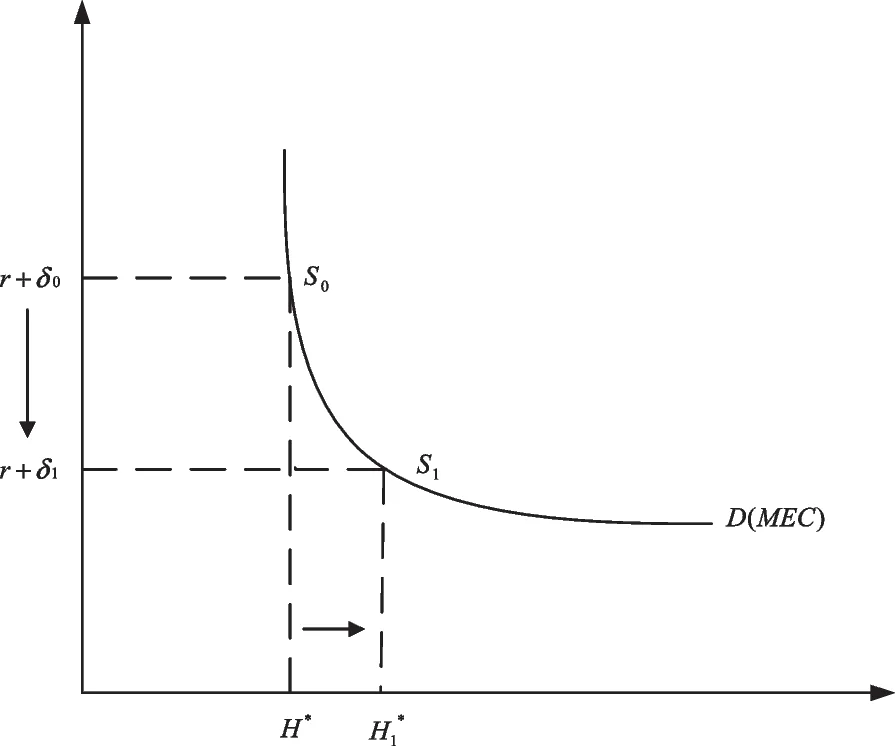
Health demand curve and supply curve

The health demand curve D and the supply curve S are plotted in Fig. 3. According to Eq. (5), when the clean heating policy improves air quality, the depreciation rate decreases, and the health supply curve shifts downward. The optimal health demand increases from H^*^ to H_1_^*^. In the context of this study, the concept of health capital (Hₜ) is extended to include cognitive health. Cognitive ability, as a manifestation of brain health, is a key 'service' derived from the health capital stock^2^.

Based on the above analysis, we propose hypothesis 2.

**Fig. 4:**
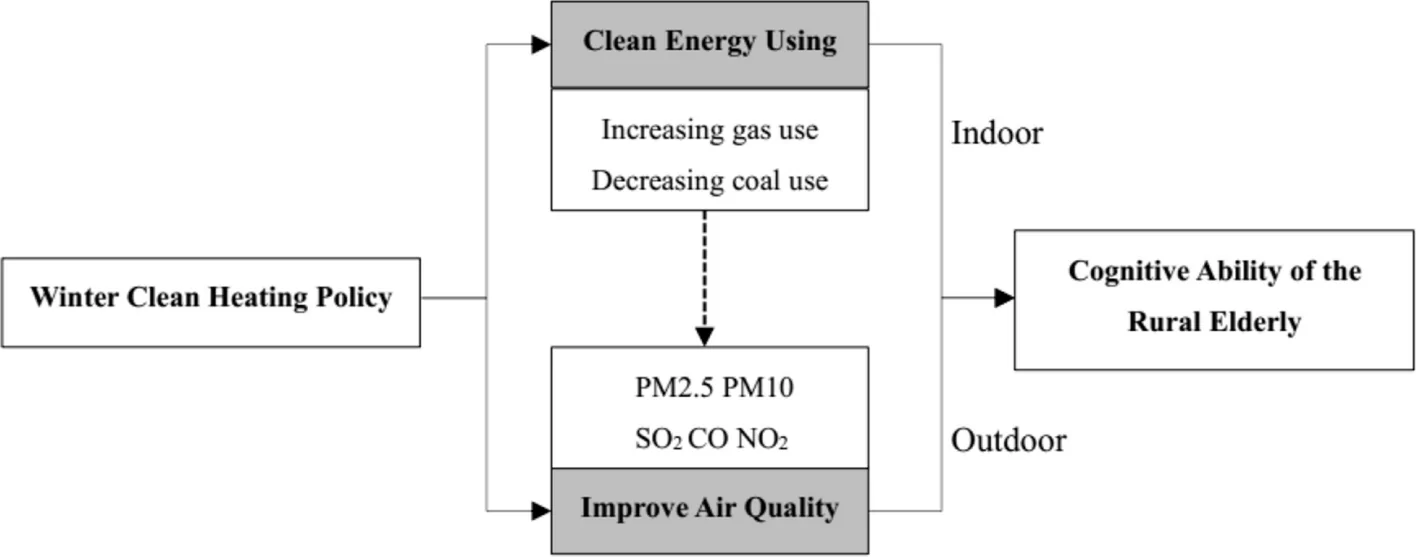
Diagram of the analytical framework

Hypothesis 2: Clean heating policies contribute to the cognitive ability of rural older people by reducing pollutant emissions (Fig. 4).

## Research design and data description

### Research design

To identify the impact of the winter clean heating pilot on the cognitive ability of rural elderly people, we treat the establishment of the pilot as an exogenous shock and employ a DID model to estimate its impact on the health of the elderly. The Staggered DID model is an extension of the traditional DID approach that is particularly useful when the treatment (policy change or intervention) is implemented at different times across different groups. This model allows us to estimate the causal effect of the treatment by comparing the changes in outcomes over time between the treated and control groups, while accounting for the staggered timing of the treatment. Following Shi, Shen, and Song [44], we set up model as

14 $${Cognitive}_{ijt}={\beta }_{0}+{\beta }_{1}{WCHP}_{jt}+\gamma {Control}_{itj}+{\mu }_{j}+{\nu }_{t}+{\varepsilon }_{itj}$$

${Cognitive}_{itj}$ is the dependent variable, which represents the health status of the ith individual in city *j* in year* t*. ${WCHP}_{jt}$ is the core explanatory variable. If a city* j* has been established as a winter clean heating pilot city in year *t*, ${WCHP}_{jt}$ takes the value of 1, otherwise it is 0. The coefficient *β*_*1*_ reflects the impact of the winter clean heating policy on the health of rural elderly. ${Control}_{itj}$ denotes a group of control variables, which specifically includes three types, namely, individual-level, household-level, and city-level. ${\mu }_{j}$ is the individual fixed effect, controlling for the characteristics that do not change over time at the individual level. ${\nu }_{t}$ denotes year fixed effect, controlling for the macro-policy shocks faced by the individuals collectively. ${\varepsilon }_{itj}$ is the random error term.

### Data sources

This paper mainly uses data from the China Health and Retirement Longitudinal Study (CHARLS) database. CHARLS is a survey data targeting individuals aged 45 and above in China, initiated by Peking University. The baseline survey started in 2011, and as of now, data from 2011, 2013, 2015, 2018, and 2020 have been released, covering 150 county-level units in China and tracking approximately 170,000 individuals from 10,000 households^3^. The CHARLS questionnaire covers information on the demographic characteristics, health status, and household energy use of middle-aged and elderly people, providing valuable data resources for this study on the health of the elderly. Since the policy implementation mainly targets the northern regions, we exclude samples from the southern regions and only retain samples from the northern regions. In addition, we also retain rural samples and the elderly population aged 60 and above. For city-level variables, we mainly obtain data from the "China Urban Construction Statistical Yearbook".

### Description of variables

1. Dependent variable, cognitive ability of the elderly. Considering that the impact of solid fuel use on health is mainly concentrated on cognitive ability and related diseases [46–50], we primarily analyze the effects of winter clean heating pilot projects on the cognitive variables. Following Cui et al. [51], we filtered two indicators from the questionnaires of previous years: (1) mental integrity, which is a composite score (0–11) based on a series of cognitive tasks including subtracting 7 from 100 to 5, naming today's date (month, day, year), recalling a day of one week, and redrawing pictures presented by the investigator. (2) situational memory score (0–10), which was measured by reading 10 nouns to the respondent and asking them to repeat the words immediately after reading them and recording the number of nouns recalled correctly^4^. we sum the two scores to obtain the "cognitive ability" variable.
2. The core explanatory variable, "Winter Clean Heating Pilot(WCHP) " is the product of the time dummy variable (Time) and the group dummy variable (Treated). The setting criterion for the time dummy variable is that the years after the city is designated as a pilot city are set to 1, while the previous years are set to 0. The setting criterion for the group dummy variable is that the cities designated as pilot cities are set to 1, while non-pilot cities are set to 0. The list of pilot cities is sourced from the government websites of the Ministry of Finance, the National Energy Administration, the Ministry of Housing and Urban–Rural Development, and the Ministry of Environmental Protection. In this paper, we match these pilot cities with the address information published in the CHARLS database on a one-by-one basis to obtain the treated group for this study. It should be noted that in the empirical analysis, we exclude all municipalities directly under the central government.
3. Control variables. Following the existing literature [44, 48, 52, 53], our control variables include: age, marital status, education^5^, whether covered by public insurance and whether receiving pension, whether co-reside with their children, frequency of smoking, and frequency of drinking. Noticed, gender, marital status, co-residence status and social insurance are dummy variables. Among them: married individuals form the observation group, while unmarried individuals form the control group; individuals with public insurance form the observation group, while those without public insurance form the control group; individuals receiving pension form the observation group, while those not receiving pension form the control group. ‘Co-reside’ is coded as 1 if at least one child co-resides, and 0 otherwise. Smoking frequency is the average number of cigarettes smoked per day now, and drinking frequency is the average number of drinks per month in the past year^6^. Descriptive statistics for each variable are shown in Table 1.

**Table 1:** Characteristics of the variables

	Control group (Treated = 0)			Experimental group (Treated = 1)		
	Average	Minimum	Maximum	Average	Minimum	Maximum
Cognitive	10.379	0	20	10.821	0	20
Age	66.263	60	100	66.059	60	91
Marriage	0.825	0	1	0.829	0	1
Education	2.800	1	8	3.342	1	9
Public health insurance	0.938	0	1	0.950	0	1
Pension	0.630	0	1	0.635	0	1
Smoking frequency	4.844	0	60	4.465	0	70
Drinking frequency	1.588	0	9	1.219	0	9
Co-reside	0.430	0	1	0.465	0	1

Comparison of data between control and experimental groups in the table is before the policy occurred (2011, 2013, 2015)

## Basic results and robustness tests

### Main results

Our benchmark results are obtained from the regression of model (14), as shown in Table 2, which examines the overall impact of the WCHP pilot on the health of rural older adults. Column (1) presents the two-way fixed effects results without adding control variables. Column (2) gradually adds other factors that may affect the cognitive ability of the elderly based on column (1). According to the regression results of columns (1) and (2), the impact of WCHP cities on the cognitive ability of rural older adults is positive and significant at the 5% level, indicating that WCHP is improving the cognitive ability of rural older adults. In terms of magnitude, the estimated effect size of 0.358 represents an improvement of approximately 3.3% relative to the pre-treatment mean of the treatment group (10.821). This conclusion constitutes a beneficial supplement to the results of previous literature studies [4, 30, 31]. Next, we further employ a series of tests to examine the robustness of the above conclusions.

**Table 2:** Baseline results for individual health

	(1)	(2)
	Cognitive	Cognitive
WCHP	0.342**	0.358**
	(0.137)	(0.136)
Age		−0.142
		(0.128)
Marriage		0.557***
		(0.165)
Education		0.003
		(0.099)
Public health insurance		0.214
		(0.222)
Pension		0.171*
		(0.097)
Smoking frequency		−0.008
		(0.013)
Drinking frequency		−0.011
		(0.026)
Co-reside		−0.027
		(0.086)
Constant	11.596***	19.757**
	(0.122)	(7.902)
Individual FE	YES	YES
Year FE	YES	YES
Number of observations	5625	5625
Adjusted R-squared	0.175	0.178

Heteroskedasticity robust standard errors clustered to the city level are in parentheses, *** denotes *p <* 0.01, ** denotes *p <* 0.05, * denotes *p <* 0.1

### Robustness tests

#### Parallel trend test

The validity of the DID model estimation results depends on whether it can pass the parallel trend test. That is, we hope that there is no significant difference in the health status of rural older adults between the treated group and the control group before the WCHP pilot. Based on the benchmark regression model, we use the event study method for the parallel trend test [54]. The specific econometric model is constructed as follows, where WCHP is a dummy variable representing the k-th questionnaire survey period implemented in the pilot cities^7^. Where βk represents the estimated value of each period. It should be noted, when using the event study method for parallel trend testing and dynamic effect analysis, to avoid the problem of multiple collinearity, it is often necessary to set a base period as a comparison object. Therefore, we use the year before the creation of the pilot city as the base period. The coefficient of βk represents the difference in the cognitive ability of rural older adults between the treated group and the control group compared with the year before the creation of the pilot city.

15 $${Cognitive}_{itj}={\beta }_{0}+{\beta }_{k} \sum\nolimits_{k=\mathrm{-2}}^{k={3}}{WCHP}_{it}^{k}+\gamma {Control}_{itj}+{\mu }_{j}+{\nu }_{t}+{\varepsilon }_{itj}$$

**Fig. 5:**
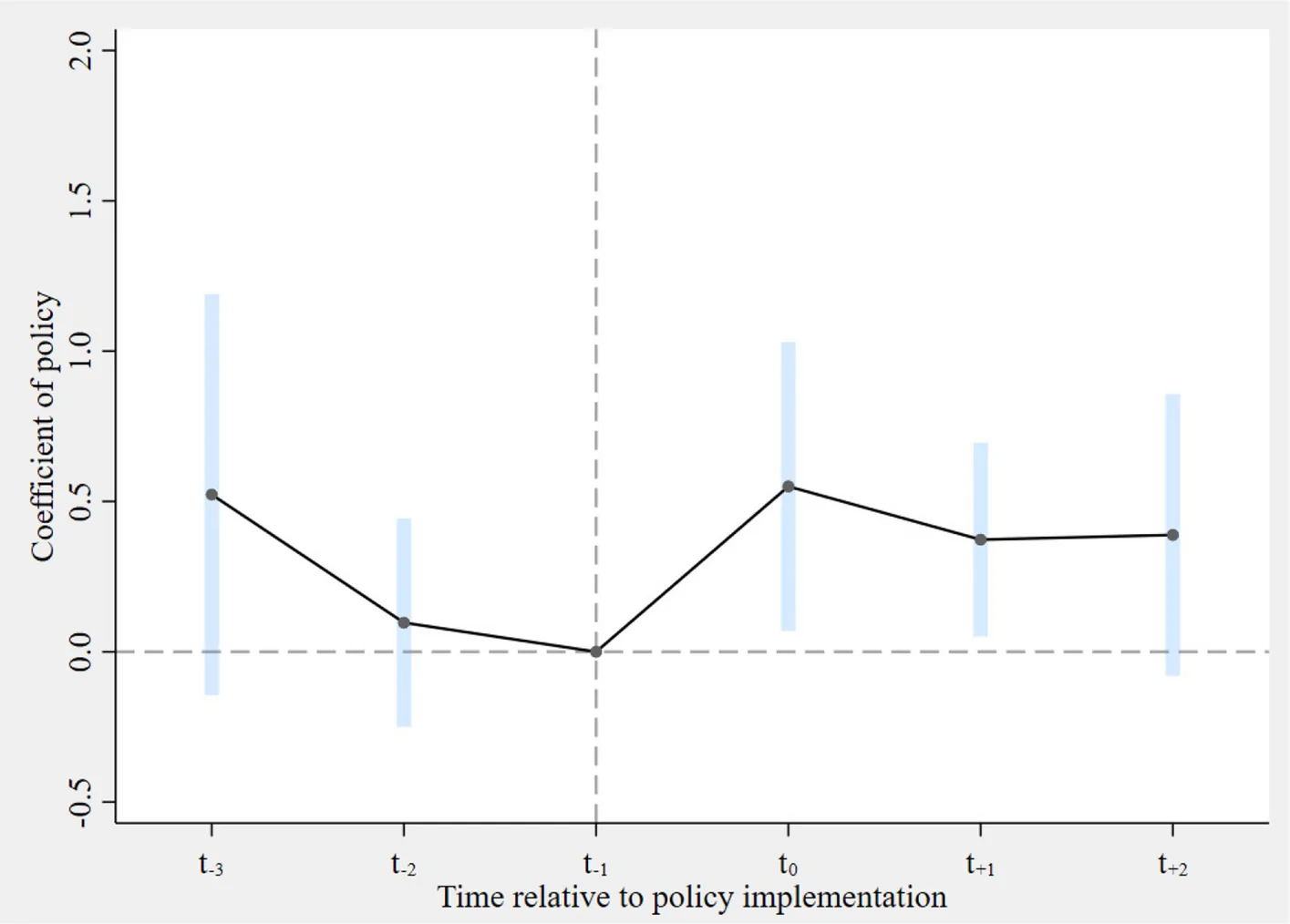
Parallel trend test (Cognitive Ability)

The results of the parallel trend test are shown in Figs. 5, corresponding to "cognitive ability" as the dependent variable. It can be found that before the creation of the WCHP city, there was no significant difference in cognitive ability between the treated group and the control group, which indicates that our results pass the parallel trend test, and the estimation results of the DID model have a certain degree of credibility. After the creation of the pilot city, there was a significant increase in cognitive ability among older adults in the treated group compared to the control group. However, the parallel trend graph shows that, the policy implementation does not last for a long time, and it only works during the period of policy implementation and the following period. The possible reason stands that the subsidy for clean energy only lasts for 3 years [55]. According to a survey conducted in Shandong Province, China, 76.4% of respondents indicated that they received investment expenditures and subsidies from policies supporting the development of renewable energy in rural areas [56], revealing the high dependence of rural residents on government subsidies. After the subsidy policy ends, rural residents who can no longer use the subsidies have reduced affordability for clean energy, which leads to a decrease in the use of clean energy and thus the health-promoting effect is no longer significant. This indicates that the observed cognitive improvement is essentially a flow benefit, highly contingent on the sustained use of clean energy, which in turn depends directly on continued economic incentives or long-term affordability.

#### Placebo test

**Fig. 6:**
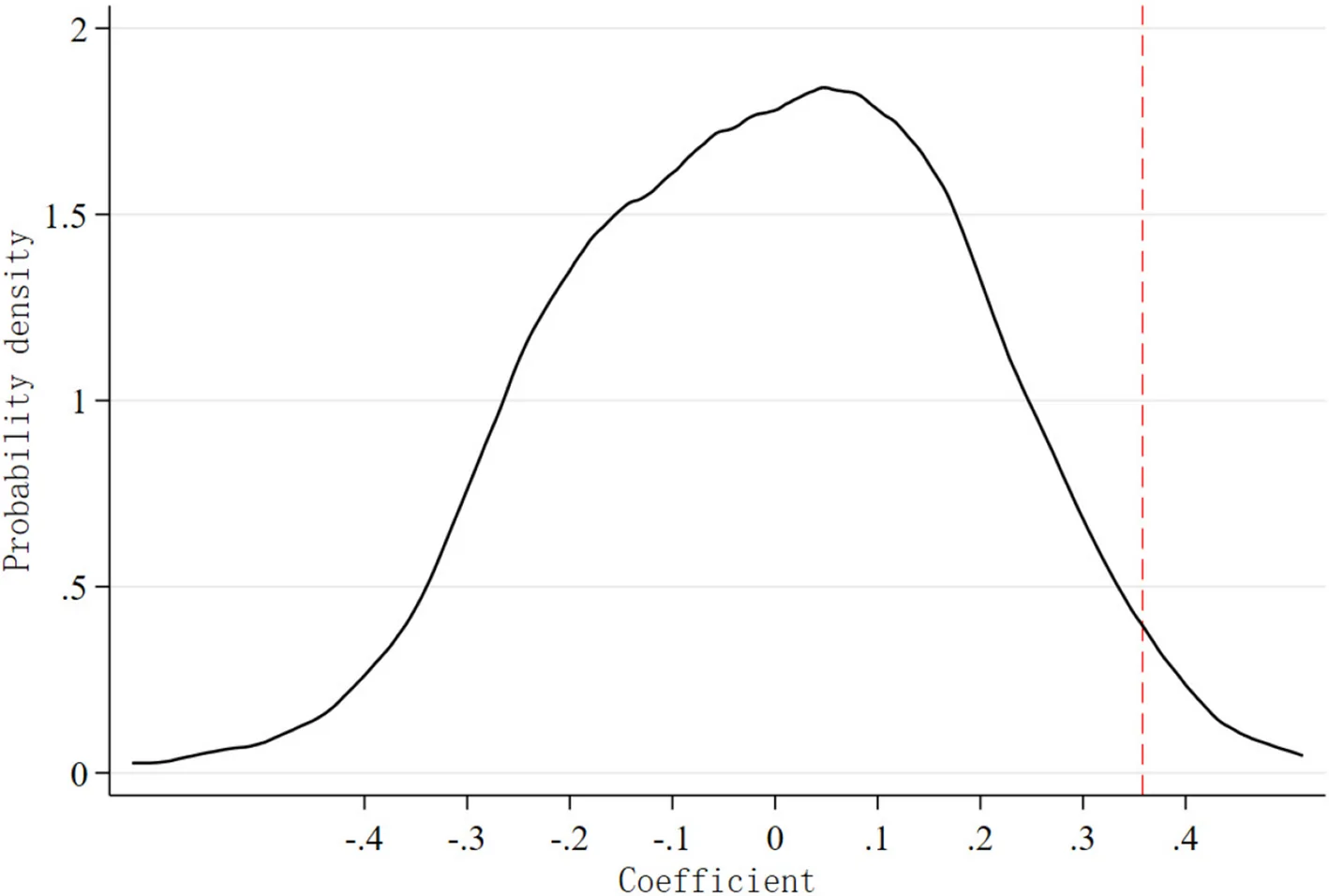
Placebo test (Cognitive Ability)

Another concern in using the DID model to assess the impact of WCHP establishment on the cognitive health of older adults is that the aforementioned conclusions may be a random phenomenon. To prevent the baseline results from being driven by some random factors, we draw on Li et al. [57] to conduct a placebo test with random sampling. The core idea of the placebo test is to falsely form the treated group or the time of the policy for estimation, and if the regression results of the estimators under different construction methods are still significant, then it indicates that the original estimation is likely to be biased, and that the dependent variables are most likely to be driven by other policies or stochasticity factors. Specifically, in each random sampling, 17 cities are randomly selected out of the 122 sample cities as a virtual treated group, and a year of the sample period was also randomly determined as the time when the WCHP cities were created. The above steps were repeated 500 times. We then plotted the kernel densities of the regression coefficients resulting from the 500 regressions in Fig. 6. By comparing them with the coefficients obtained from the aforementioned benchmark regression (whose positions are indicated by red dashed lines in the figure), the results show that the estimated coefficients for most of the dummy regressions are concentrated around 0, and the baseline regressions are unlikely to be random. This further suggests that the creation of the WCHP cities has improved the cognitive ability of the treated group, and that the findings of the previous study are plausible.

#### Omitted variable problem

Another potential issue is that the relationship between WCHP establishment and the cognitive health of rural residents may also be co-driven by other omitted variables, such as the level of green area and healthcare conditions. Therefore, we further control for the greening rate and the number of hospitals in the cities where the sample residents are located, where urban greening is measured by the ratio of the urban green space area to the built-up area, and the data are obtained from the China Urban Statistical Yearbook. The estimation is also based on model (14), and the coefficient of the interaction term remains significant, indicating that the establishment of WCHP still improves the mental and physical health of the rural elderly^8^.

In addition, following Altonji et al. [58], we also adopt the boundary test method to examine the endogeneity problem caused by the possible omission of important unobservable variables. First, constructing two different sets of control variables, one controlling only a limited number of variables and the other controlling all variables. Then, adding these two sets of control variables to the model separately to run the regression, for which the estimated coefficient of the core explanatory variables controlling the limited variables are denoted as β^R^, and the coefficient of the core explanatory variables controlling all variables are denoted as β^F^, We can construct the following indicators:

16 $$Ratio=\left|\frac{{\beta }^{F}}{{\beta }^{F}-{\beta }^{R}}\right|$$

The larger the Ratio obtained through Eq. (16), the greater the explanatory power of the variables of interest that have been controlled, and the smaller the effect of the omitted variables. In our study, four sets of variables are constructed: no control variables, controlling for individual-level variables, controlling for city-level variables, and controlling for all variables. The first three sets of variables are used as the finite set of control variables, and we obtain three Ratios. Based on the core regression coefficient with 'cognitive ability' as the dependent variable, the minimum value we obtain is 6.974, and the mean value is 11.390. This result indicates that only when the explanatory power of the omitted variable is at least 6.974 times that of all control variables and, on average, more than 11.390 times, can the omitted variable have an impact on the benchmark estimation results. Thus, the main conclusions of this paper remain robust to the omitted variable problem.

#### Exclusion of other relevant policies in the same period

During the period of WCHP, there are indeed three other related policies that may have an impact on the health of middle-aged and elderly people. First, the "Healthy City" pilot policy. This policy was implemented in 2016, which coincides with the establishment period of WCHP cities, and aims to improve the health literacy of the residents [59], which may affect the benchmark estimation results. Second, the long-term care insurance pilot. In June 2016, the Ministry of Human Resources and Social Security issued a document selecting 15 cities and 2 provinces across the country to carry out long-term care insurance system pilots. The pilot cities can affect the cognitive health of middle-aged and elderly people by promoting the development of the elderly care service industry [60]. Third, the Ecological Civilization Construction pilot policy, implemented during the same period, aims to advance regional green development and ecological protection [61] and may indirectly influence the health of middle-aged and older adults through overall environmental improvements. To rule out the interference of these three policies on the estimation results, we control for the corresponding policy dummy variables in the model and re-estimate the specifications. The results, presented in columns (1) to (3) of Table 9 in the Appendix, show that the estimated coefficient of the WCHP remains robust.

#### Heterogeneous treatment effects

To address the negative weighting problem in staggered DID models, following the method of De Chaisemartin and D'Haultfoeuille [62], we try to solve the two-way fixed-effects DID estimator (TWFEDD) under the heterogeneous treatment condition. The results show that using "cognitive ability" as the dependent variable generates 225 positive weights and only 2 negative weights out of 227 ATTs, indicating that the heterogeneity treatment effect did not have a substantial impact on the baseline results and that the regression coefficients were plausible.

We also apply another approach that effectively addresses the potential "negative weights" issue. JWDID represents an advancement on the traditional two-way fixed effects (TWFE) model by employing a two-way Mundlak specification [63]. As presented in Table 10 in Appendix, the estimated coefficient for the policy variable under the JWDID estimator remains positive, statistically significant, and quantitatively close to our baseline TWFE results. This consistency strongly suggests that our main findings are not substantially biased by the negative weights problem.

To ensure the robustness of our cognitive outcome measurement, we complemented our analysis with an alternative, well-validated measure less sensitive to these biases: immediate recall in a situational recall task [52, 64]. Specifically, we used a 10-noun immediate recall task. The interviewer read a list of 10 nouns aloud, and respondents were asked to repeat as many as they could remember immediately after presentation. The score—the number of correctly recalled words—serves as a direct measure of episodic memory, a core component of cognitive function that is less dependent on literacy, vision, or cultural familiarity with specific objects. As shown in Columns (1)-(2) of Table 11 in Appendix, the estimated effect of the clean heating policy remains positive and statistically significant (*p <* 0.05) when using this recall-based measure. This consistency across measurement approaches suggests that our main finding is not driven by potential biases in other cognitive subtests, and reinforces the robustness of the result to alternative operationalizations of cognitive status.

## Analysis of mechanisms

### Clean energy use

Previous theoretical analysis shows that, through subsidies, WCHP reduces the price of clean energy use, thereby increasing the likelihood of households using clean energy. Based on a large-scale survey of rural households in northern China, Wang and Xie [65] finds that both energy subsidies and mandatory measures effectively contribute to the decision of rural households to shift from coal to electricity and natural gas for heating energy. In addition, after the implementation of the policy, each village also adopted unique propaganda methods to persuade households to participate in the clean heating project, such as holding villagers' meetings, broadcasting, home visits, and bulletin boards. We incorporate the following four variables to validate the above transmission paths, containing whether the household has a natural gas supply, whether it has collective heating, whether it is heated by coal and whether it uses coal as the main source of cooking. From the estimation results in Table 3, it can be seen that the WCHP significantly improves the accessibility of clean energy to rural households, inducing households to use natural gas and reducing the use of solid fuels^9^. According to the existing literature, clean energy use can directly improve the health of household members [1, 30, 31, 46]. Thus, we validate research hypothesis 1, which states that policies can improve the cognitive health of household members by increasing the household's use of clean energy.

**Table 3:** Channel I: clean energy use

	(1)	(2)	(3)	(4)
	Gas supply	Communal heating	Coal-fired heating	Cooking with coal
WCHP	0.074**	0.040***	−0.121***	−0.215***
	(0.029)	(0.012)	(0.020)	(0.025)
Controls	YES	YES	YES	YES
Constant	0.037***	0.045***	0.965***	0.786***
	(0.013)	(0.007)	(0.006)	(0.012)
Household FE	YES	YES	YES	YES
Year FE	YES	YES	YES	YES
Number of observations	6830	6828	3619	6788
Adjusted R-squared	0.021	0.008	0.087	0.056

Heteroskedasticity robust standard errors clustered to the city level are indicated in parentheses in the table, *** indicates *p <* 0.01, ** indicates *p <* 0.05, and * indicates *p <* 0.1

### Outside air quality

According to the previous theoretical model, clean heating policies can contribute to the health of rural elderly by reducing pollutant emissions. To validate our analysis, following [2], including PM2.5, PM10, SO_2_, CO, and NO_2_ as proxy variables for air pollution. These pollutants are highly correlated with solid fuel emissions and also to have a significant negative impact on the health of the population. Since these variables are city-level, we construct panel data (2011–2020) by collecting other city-related information to keep all variables on the same dimension so as to perform two-way fixed effects estimation. The results in Table 4 show that the WCHP significantly reduces the level of pollutant emissions and improves air quality in pilot area. Thus, hypothesis 2 is verified. It should be noted that we do not suggest that the two current mechanisms are in a parallel relationship. More likely, the WCHP improves cognitive health outcomes by increasing the use of clean energy, which in turn improves indoor and local air quality.

**Table 4:** Channel II: outside air quality

	(1)	(2)	(3)	(4)	(5)
	PM2.5	PM10	SO2	CO	NO2
WCHP	−5.944***	−6.783**	−16.341***	−0.350***	−1.849*
	(1.781)	(3.014)	(1.953)	(0.039)	(0.994)
Controls	YES	YES	YES	YES	YES
Constant	103.401***	132.806***	22.054	2.008**	54.013***
	(24.598)	(41.588)	(25.451)	(0.801)	(11.864)
City FE	YES	YES	YES	YES	YES
Year FE	YES	YES	YES	YES	YES
Observations	2,221	2,221	2,221	2,221	2,221
Adjusted R-squared	0.739	0.745	0.736	0.676	0.820

Standard errors are in parentheses. *** indicates *p <* 0.01, ** indicates *p <* 0.05, * indicates *p <* 0.1. Air pollution indicators are from the China Meteorological Observation Platform. Control variables include: log of the number of patents, log of per capita GDP, level of inclusive financial development, level of green financial development, log of science and technology expenditures, log of education expenditures, level of industrial structure, and log of total consumption

## Extensions

### Heterogeneity in different groups

#### Differences in age group

We further explore whether there is a heterogeneous response between the middle-aged group and older age groups. So we classify the age between 45–60 years old as the middle-aged group. The sub-sample estimation results are in Table 5, showing that the WCHP effect is only significant for the older age group, but not for the middle-aged group. The possible reason is that the middle-aged group is more resistant and resilient than the older group, and has better lung function, resulting in less injury from solid fuel pollutants. In contrast, the older age group is in relatively poorer physical condition and has higher vulnerability to solid fuel pollutants, thus benefiting more from clean energy transition policies.

**Table 5:** Heterogeneity across age groups

	Cognitive	
	Middle age	Older
	(1)	(2)
WCHP	−0.108	0.358**
	(0.171)	(0.136)
Controls	YES	YES
Constant	11.527***	19.783***
	(3.939)	(7.887)
Individual FE	YES	YES
Year FE	YES	YES
Number of observations	8597	5625
Adjusted R-squared	0.115	0.178

Heteroskedasticity robust standard errors clustered to the city level are in parentheses

^***^ indicates *p <* 0.01, ** indicates *p <* 0.05, * indicates *p <* 0.1

#### Differences in gender

Relevant studies have found that late-life exposure to ambient air pollution may accelerate cognitive aging in middle-aged and older adults, with significant gender disparities observed—women demonstrating higher vulnerability to these adverse effects [66]. We further explore whether there is a heterogeneous response between the male group and female groups. The sub-sample estimation results are in Table 6. The regression coefficient for female is higher than that for male and is statistically significant, suggesting that WCHP has a greater impact for females on the cognitive ability. The results suggest that large-scale clean energy transitions can help mitigate gender disadvantages, particularly in the Chinese context where women disproportionately shoulder energy-intensive domestic labor.

**Table 6:** Heterogeneity across gender groups

	Cognitive	
	Male	Female
	(1)	(2)
WCHP	0.140	0.687***
	(0.151)	(0.244)
Controls	YES	YES
Constant	38.421***	−14.810
	(10.030)	(11.817)
Individual FE	YES	YES
Year FE	YES	YES
Number of observations	3128	2497
Adjusted R-squared	0.192	0.172

Heteroskedasticity robust standard errors clustered to the city level are in parentheses

^***^ indicates *p <* 0.01, ** indicates *p <* 0.05, * indicates *p <* 0.1

#### Differences in educational attainment

According to the Health Behavior Gradient and Fundamental Cause Theory [67], socioeconomic status (SES) is a "fundamental cause" of health disparities because it embodies resources—such as knowledge, money, power, and social connections—that can be deployed to avoid health risks. Lower education, a key component of SES, limits these resources. Zhu et al. [33] found that the level of education of the household head and the accessibility and affordability of energy are important factors for household cooking energy choices. So we divide the sample according to whether the respondents have a primary school diploma or not. As shown in Table 7, the results show that the policy has more significant effect for the less educated rural dwellers, better improving the cognitive ability in this group. This may be due to the fact that this group has lower income and higher affordability of clean energy before the policy implementation. In addition, the highly educated group is more health-conscious and thus higher standard on fuel emissions. This pattern aligns closely with the socioeconomic gradient in health framework. Therefore, the impact of the post-policy was significant among the less educated groups, who were less health-conscious and favored cost-saving energy sources, and therefore used solid fuels more often. The results suggest that the "most vulnerable" of the disadvantaged groups have improved their well-being.

**Table 7:** Heterogeneity in educational attainment

	Cognitive	
	Low education	High education
	(1)	(2)
WCHP	0.473***	0.165
	(0.171)	(0.255)
Controls	YES	YES
Constant	10.897	32.721***
	(10.811)	(11.913)
Individual FE	YES	YES
Year FE	YES	YES
Number of observations	4101	1524
Adjusted R-squared	0.183	0.177

Heteroskedasticity robust standard errors clustered to the city level are in parentheses

^***^ indicates *p <* 0.01, ** indicates *p <* 0.05, * indicates *p <* 0.1

#### Whether living with children

We further explore whether policy effects are heterogeneous across families with different living conditions. Generally speaking, family members share similar health knowledge and values, and thus children may influence their parents' health habits. Evidence shows that elderly parents may receive economic resources, cleaner fuels, and improved sanitation from their children, leading to improved physical health [68]. In this paper, we divide the sample according to whether the respondents lived with their children or not. As shown in Table 8, the policy effect is more effective for the rural elderly living with their children, including promotion of cognitive ability (significant at the 1% level). This results imply that cohabitation with children can promote household clean energy transition through health literacy, which in turn improves the health of elderly parents. In contrast, the WCHP did not have a significant impact on ‘empty nesters’. The possible reasons lie that elderly parents who do not live with their children usually accompanied with weaker health awareness, and also they are limited to economic resources, resulting in a lower affordability of energy.

**Table 8:** Whether children co-living with respondent

	Cognitive	
	Co-Reside	No Co-Reside
	(1)	(2)
WCHP	0.873***	0.197
	(0.229)	(0.245)
Controls	YES	YES
Constant	7.406	31.797*
	(11.853)	(18.124)
Individual FE	YES	YES
Year FE	YES	YES
Number of observations	2312	3264
Adjusted R-squared	0.214	0.182

Heteroskedasticity robust standard errors clustered to the city level are in parentheses

^***^ indicates *p <* 0.01, ** indicates *p <* 0.05, * indicates *p <* 0.1

### Impact on household medical expenditure

We also wonder whether the WCHP (Winter Clean Heating Program) policy can reduce medical expenditures by improving the cognitive health of rural residents. Specifically, we take the logarithm of household medical expenses in the past year as the dependent variable and use panel Tobit estimation to address the issue of a large number of 0 values in the dependent variable. The regression results are shown in Table 14 in Appendix. Whether or not we control for household head-related variables, the WCHP coefficient is negative at the 10% significant level, indicating that this policy can reduce household medical expenditures and play a role in alleviating the phenomenon of "poverty due to illness" in rural areas. This conclusion is consistent with the findings of some related literature [29, 69].

## Research findings and discussions

We try to elucidate the effects of clean energy policies on the health of the elderly through the construction of a theoretical model. Based on the 5-wave longitudinal survey data of CHARLS, we use the DID method to explore the impact of the "winter clean heating" policy implemented in northern regions on the health of the elderly in rural China, using cognitive ability as measurement. Our research results emphasize the significant impact of clean energy transformation on the cognitive health of these groups. This conclusion remains valid after a series of robustness tests, including parallel trend tests, placebo tests, PSM-DID tests, as well as ruling out other policy interferences. The clean energy policy has a positive effect on the cognitive health of the elderly by increasing the likelihood of using clean energy and improving local air quality, which is consistent with the assumptions of the theoretical model. Our heterogeneity analysis shows that the impact of clean energy policies on the elderly is more significant than on middle-aged people. The promotional effect of clean energy transformation policies is more significant for women and educationally disadvantaged groups. In addition, the effect of health improvement is particularly significant for the elderly living with their children, implying the "sharing" of different family members' health awareness within the household. Finally, we also find that the policy has reduced the medical expenses of rural families, reflecting the "preventive" policy benefits.

Against the backdrop of an aging society, a deep analysis of the impact of clean energy transformation on the cognitive health of the elderly has profound policy implications for ensuring the welfare of rural elderly, and promoting healthy and positive aging. First, we should accelerate the 'expansion' of the 'winter clean heating' pilot program, explore the establishment of a dynamic adjustment mechanism for the pilot list, and fully promote the transformation of clean energy. New regions should be selected based on a combination of high baseline air pollution, aged population density, and household energy poverty indicators. In addition, differentiated energy subsidy standards should be implemented according to household economic conditions. On the premise of ensuring fair distribution of household energy and minimizing unfairness, the policy benefits should be maximized. Empirical analysis in this paper and related case studies show that there is an uneven distribution of resources in energy subsidy policies [70], and the current policy does not yet reflect sustainable characteristics. Therefore, based on the experience of pilot construction, we should further implement sliding-scale subsidy schemes based on verifiable household income/wealth data and the educational attainment of elderly residents.

Regarding generalizability, pilot cities self-selected based on pollution and fiscal capacity. While our estimated effect size may not extend to cities with vastly different conditions, the findings retain policy relevance. The selection prioritized high-pollution, mid-capacity cities—precisely where clean heating is most needed—as scalable test cases for national rollout. Thus, the core insight that well-structured clean heating benefits cognitive health in solid-fuel-reliant settings remains valid for comparable urban contexts.

Our research also has several limitations. First, CHARLS is a nationally representative survey targeting the Chinese population aged 45 and above. However, households without middle-aged or elderly people (children and young people) are not included in the sample and are therefore excluded from the analysis of health outcomes in this study. This also opens up the possibility of future analysis of policy impacts through other data sources. Secondly, while outdoor air quality is examined as a potential mechanism, indoor air quality—particularly exposure to pollutants from heating sources—may also play an important role in cognitive health. Due to the lack of direct measurements of indoor air pollution in the survey data, we could not test this pathway explicitly and instead relied on city-level outdoor air quality as a proxy. Future studies with more detailed indoor exposure data would help clarify this mechanism.

## Data Availability

Publicly available datasets were analyzed in this study. This data can be found here: China Health and Retirement Longitudinal Study (CHARLS) [http://charls.pku.edu.cn/] (http://charls.pku.edu.cn). Clinical trial number: not applicable.

## Appendix

**Table 9:** Table A.1 Regression results by excluding the relevant policies

	(1)	(2)	(3)
	Cognitive	Cognitive	Cognitive
WCHP	0.358**	0.357**	0.376**
	(0.137)	(0.140)	(0.141)
Controls	YES	YES	YES
Constant	19.759**	19.760**	19.123**
	(7.935)	(7.907)	(8.090)
Individual FE	YES	YES	YES
Year FE	YES	YES	YES
Number of observations	5625	5625	5470
Adjusted R-squared	0.178	0.178	0.174

Controls in columns (1)-(3) contain individual control variables as well as the implementation of the policies

**Table 10:** Table A.2. Regression results of JWDID

	(1)	(2)
	Cognitive	Cognitive
WCHP	0.328**	0.262*
	(0.149)	(0.151)
Controls	No	YES
Individual FE	YES	YES
Year FE	YES	YES
Number of observations	5160	5160
Adjusted R-squared	0.585	0.762

Columns (1)-(2) in the table indicate robust standard errors in parentheses (which are reported below), *** indicates *p*<0.01, ** indicates *p*<0.05, * indicates *p*<0.1

**Table 11:** Table A.3 Robustness check: Replacing the dependent variable

	(1)	(2)
	Cognitive	Cognitive
WCHP	0.200**	0.194**
	(0.082)	(0.082)
Controls	NO	YES
Constant	3.904***	5.925
	(0.071)	(3.801)
Individual FE	YES	YES
Year FE	YES	YES
Number of observations	8410	8410
Adjusted R-squared	0.162	0.166

Heteroskedasticity robust standard errors clustered to the city level are in parentheses, *** denotes *p*<0.01, ** denotes *p*<0.05, * denotes *p*<0.1

**Table 12:** Table A.4 Impact on different heating energy sources

	(1)	(2)	(3)
	Heating with natural gas	Heating with petroleum gas	Heating with electric
WCHP	0.054***	0.002	0.063***
	(0.011)	(0.003)	(0.018)
Controls	YES	YES	YES
Constant	0.005**	0.006**	0.022***
	(0.002)	(0.003)	(0.005)
Household FE	YES	YES	YES
Year FE	YES	YES	YES
Number of observations	3621	3621	3621
R-squared	0.039	0.002	0.044

The explanatory variables are all dummy variables. The sample size of customers using solar heating is too small to be analyzed. Heteroskedasticity robust standard errors clustered to the city level are indicated in parentheses in the table, *** indicates *p*<0.01, ** indicates *p*<0.05, and * indicates *p*<0.1

**Table 13:** Table A.5 Impact on different cooking energy sources

	(1)	(2)	(3)	(4)
	Cooking with natural gas	Cooking with petroleum gas	Cooking with electric	Cooking with marsh gas
WCHP	0.064***	0.047***	0.067***	0.003
	(0.013)	(0.012)	(0.017)	(0.002)
Controls	YES	YES	YES	YES
Constant	0.040***	0.058***	0.107***	0.006**
	(0.006)	(0.007)	(0.010)	(0.002)
Household FE	YES	YES	YES	YES
Year FE	YES	YES	YES	YES
Number of observations	6835	6835	6835	6835
R-squared	0.027	0.012	0.033	0.002

The explanatory variables are all dummy variables. Heteroskedasticity robust standard errors clustered to the city level are indicated in parentheses in the table, *** indicates *p*<0.01, ** indicates *p*<0.05, and * indicates *p*<0.1

**Table 14:** Table A.6 Impact on household medical expenditure

	(1)	(2)
	Medical expenditure	Medical expenditure
WCHP	-0.246*	-0.283**
	(0.138)	(0.138)
Household Head Controls	NO	YES
Constant	0.993***	-0.683
	(0.456)	(0.647)
City FE	YES	YES
Year FE	YES	YES
Number of observations	6996	6870

1. Since the dependent variable lagged by one period, we also lag the data year by one period. 2. The household head control variables include age, marital status, education level, whether they are covered by public insurance, and whether they receive a pension

## Footnotes

1 New WHO Global Air Quality Guidelines aim to save millions of lives from air pollution. https://www.who.int/news/item/22-09-2021-new-who-global-airquality-guidelines-aim-to-save-millions-of-lives-from-air-pollution.

2 A growing body of biomedical evidence elucidates the pathways through which air pollution impairs cognitive function, bridging our theoretical model with physiological mechanisms [42, 43]. Key pathways include: (1) Systemic and neuroinflammation; (2) Oxidative stress; (3) Cerebrovascular damage and blood-brain barrier disruption; (4) Direct neurotoxicity.

3 For more information about this data, please refer to Zhao et al. [45].

4 The situational memory scores of the first three waves of the survey (2011, 2013, and 2015) also required them to repeat the words again based on recall four minutes after reading the nouns, but since the respondents in the fourth and fifth waves of the survey deepened their memories by the investigator repeating the words three times before delaying recall, we excluded the delayed memory score indicator.

5 According to the original questionnaire's assignment method, no schooling = 1, primary school = 2, junior high school = 3, senior high school = 4, vocational high school = 5, technical school = 6, secondary school = 7, tertiary school = 8, bachelor's degree = 9, and master's/doctoral degree = 10.

6 None = 0, less than once a month = 1, once a month,2, 2-3 times a month = 3, once a week = 4, 2-3 times a week = 5, 4-6 times a week = 6, once a day = 7, twice a day = 8, more than twice a day = 9.

7 for example, for the 2017 policy, the 2015 questionnaire corresponds to the first period before the policy implementation, and the 2018 questionnaire corresponds to the first period after the policy implementation

8 Due to space limitations, the results are left in the appendix.

9 Tables 12 and 13 in the Appendix show that the policy has led to a greater shift in the use of electricity, petroleum gas and natural gas as daily energy sources.
